# Localization Performance in a Binaural Real-Time Auralization System Extended to Research Hearing Aids

**DOI:** 10.1177/2331216520908704

**Published:** 2020-04-23

**Authors:** Florian Pausch, Janina Fels

**Affiliations:** Teaching and Research Area of Medical Acoustics, Institute of Technical Acoustics, RWTH Aachen University

**Keywords:** virtual acoustic environments, sound source localization, binaural technology, hearing aids

## Abstract

Auralization systems for auditory research should ideally be validated by perceptual experiments, as well as objective measures. This study employed perceptual tests to evaluate a recently proposed binaural real-time auralization system for hearing aid (HA) users. The dynamic localization of real sound sources was compared with that of virtualized ones, reproduced binaurally over headphones, loudspeakers with crosstalk cancellation (CTC) filters, research HAs, or combined via loudspeakers with CTC filters and research HAs under free-field conditions. System-inherent properties affecting localization cues were identified and their effects on overall horizontal localization, reversal rates, and angular error metrics were assessed. The general localization performance in combined reproduction was found to fall between what was measured for loudspeakers with CTC filters and research HAs alone. Reproduction via research HAs alone resulted in the highest reversal rates and angular errors. While combined reproduction helped decrease the reversal rates, no significant effect was observed on the angular error metrics. However, combined reproduction resulted in the same overall horizontal source localization performance as measured for real sound sources, while improving localization compared with reproduction over research HAs alone. Collectively, the results with respect to combined reproduction can be considered a performance indicator for future experiments involving HA users.

Extensive research in acoustic virtual reality (Vorländer, 2007) and increasing computational power have enabled the flexible generation of virtual acoustic environments (VAEs) to recreate complex auditory scenes (Bregman, 1994; Virtanen et al., 2018) in real time (Schröder, 2011; Wefers, 2015). In auditory research involving people with hearing loss (HL) fitted with hearing aids (HAs), reproduction of VAEs via headphones is not feasible, since most HA algorithms depend on acoustic cues from spatially distributed sound sources and the acoustic environment itself to work properly. A loudspeaker-based spatial audio reproduction is therefore necessary and raises the questions as to which approach should be used, given the typical advantages and limitations of each technology (Pausch et al., 2018; Spors et al., 2013), and how to properly integrate HAs into the virtual scene. Various spatial audio reproduction systems have been applied by different groups (see, e.g., Cubick & Dau, 2016; Grimm et al., 2016; Oreinos & Buchholz, 2016; Seeber et al., 2010) for HA-related research. As studies in this area should preferably be conducted without being confined to specific HA manufacturers or models, the researchers must have access to the parameter settings of HA algorithms. Using commercially available HAs with only partially controllable proprietary algorithms, or even different models across participants will likely lead to biased experimental results. In general, full control over simulation and playback signals is therefore a crucial feature of any auralization system used for auditory experiments to facilitate reproducibility.

Based on these requirements, a loudspeaker-based binaural real-time auralization system was extended by an interface to research HAs that grant access to raw microphone and HA receiver signals (Pausch et al., 2018). The system facilitates measuring HA-related transfer functions (HARTFs) on a spatial grid (e.g., Denk et al., 2018; Kayser et al., 2009; Oreinos & Buchholz, 2013; Thiemann & van de Par, 2019), which are subsequently utilized for the generation of binaural signals, optionally in combination with room acoustic simulations (Schröder, 2011). Prior to playback, the HA signals are additionally processed on a master HA, a real-time software platform (e.g., Curran & Galster, 2013; Grimm et al., 2006; Herzke et al., 2017), emulating conventional HA algorithms which can be customized to individual audiograms given full parametric control. The proposed system was designed for users with mild to moderate HL. This aspect was considered not only by reproducing signals over research HAs but also via loudspeakers in combination with acoustic crosstalk cancellation (CTC) filters (Atal et al., 1966; Masiero, 2012) for external sound field reproduction. Binaural signals used in the loudspeaker-based playback path are dependent on measured generic, individual, or individualized head-related transfer functions (HRTFs). Both auralization paths are consolidated in an HA auralization module with time alignment option by means of a variable delay line, simulating real-life HA delays (Stone et al., 2008). To allow for user interactivity, an optical tracking system is integrated to capture real-world user movements, triggering filter updates. Due to low hardware requirements, the proposed setup can be installed in rooms with limited space such as hearing booths. A detailed description of the specific system implementation including an objective evaluation of system components, a simulation benchmark analysis, and end-to-end latency measurements is provided in Pausch et al. (2018).

For a more complete evaluation of auralization systems, objective experiments should be complemented by perceptual ones. Although there are auditory models that predict perceptual parameters (Baumgartner et al., 2014; Nowak & Klockgether, 2017), individual differences in spatial audio reproduction systems (e.g., underlying concepts, system implementations, etc.) and particular research questions render it necessary to conduct specifically designed listening experiments focusing on selected spatial audio quality inventory parameters (see, e.g., Lindau et al., 2014; Nicol et al., 2014; Raake et al., 2014; Simon et al., 2016). Among these parameters, the localization of sound sources is a crucial one for systems replicating real-life acoustic environments by means of VAEs. It is well known that binaural cues like interaural level and time differences (Blauert, 1997; Møller et al., 1995; Rayleigh, 1907) as well as monaural cues, that is, spectral filter characteristics in higher frequency regions occurring due to pinna resonances, contribute to source localization. The latter are especially helpful to determine sources lying on cones of confusion and for elevated sound sources (Musicant & Butler, 1985; Wightman & Kistler, 1997). In addition to static cues, dynamic binaural cues can be accessed through head movements, which further improve localization by reducing reversal rates (Bomhardt & Fels, 2017; McAnally & Martin, 2014; Thurlow & Runge, 1967).

Since it is unclear how well these localization cues are retained by the system under consideration (Pausch et al., 2018), we investigated localization performance when using its individual reproduction paths and the combination thereof. Results are discussed with respect to the baseline conditions for virtual sound source (VSS) and real sound source (RSS) localization using headphones and discrete loudspeaker playback, respectively. The study aimed at answering two main research questions: How does localization performance differ when playing back VSSs dynamically over headphones, loudspeakers with CTC filters, research HAs alone, or combined via loudspeakers with CTC filters and research HAs, compared with playback of RSSs over discrete loudspeakers? Does binaural loudspeaker-based playback with CTC filters have an observable positive effect on localization when reproducing VSSs combined via loudspeakers with CTC filters and research HAs compared with playback over research HAs alone?

It should be clarified if the simulation can recreate real-world listening with respect to the conveyance of localization cues. Seen from a broader perspective, these investigations are important in the context of establishing a perceptual performance baseline for further evaluations. With this regard, the system may be useful for the development of standardized procedures to evaluate HA algorithms in devices with open fitting and novel fitting routines in dynamically reproduced VAEs.

## The Current Study

The focus of the study lies on investigating localization effects attributed to different reproduction systems as well as the influence of dynamic binaural cues. The experimental conditions of the dynamic localization experiment conducted in the study are outlined below.

To assess the potential of VAEs, VSSs had to be localized under free-field conditions and were compared with RSS localization, modeled by spatially distributed loudspeakers (Bronkhorst, 1995). The VSSs were either played back via headphones or loudspeakers with CTC filters based on dynamic binaural synthesis.

Since VAEs can be used as a test and training environment for users with HL (Cameron & Dillon, 2008, 2011), localization performance via HAs needs to be assessed separately. Denk et al. (2018) objectively investigated monaural cue preservation across different HA device styles in generic and individual HRTFs by applying auditory models for sagittal plane localization (Baumgartner et al., 2014). Mueller et al. (2012) tested localization ability of adults with normal hearing (NH) in realistic acoustic scenes when playing back simulated HA signals based on HARTFs over open-fit behind-the-ear (BTE) HAs. We replicated a similar playback condition under free-field conditions, in which participants had to localize VSSs reproduced via BTE receiver-in-the-ear HAs in omnidirectional mode.

Although localization performance in static CTC systems with matched and mismatched configurations, has been examined by Majdak et al. (2013), it is unknown how combined binaural playback over loudspeakers and research HAs affects localization in dynamic binaural reproduction. Hence, in addition to the localization of VSSs over loudspeakers with CTC filters and research HAs alone, we evaluated localization performance using combined reproduction.

Results are analyzed through linear mixed-effects (LME) models, predicting the overall horizontal source localization as per participants’ estimations. This part of the analysis is specifically tailored to the horizontal plane, the region most frequently used in experiments testing speech-in-noise perception or related metrics such as spatial release from masking (see, e.g., Cameron & Dillon, 2011; Ozimek et al., 2013), representing one main application area of the system. Additional analyses compare reversal rates as well as angular error metrics across experimental conditions, including sound sources on the horizontal and median planes.

## Methods

### Participants

Fifteen nonexpert adults (9 females) with self-reported NH, no history of HL, and normal (or corrected-to-normal) vision at the age of 24 ± 5.4 (mean [*M*] ± standard deviation [*SD*], range: 18–35) participated in the study. All participants provided written informed consent and were paid for their participation. The collected personal data and experimental results were processed and archived in accordance with country-specific data protection regulations.

### Stimulus Material

As stimulus, a two-pulse white noise train with unwindowed on- and offsets and a total duration of 2.25 s with an intermediate pause of 0.25 s was used. The single-channel audio file was generated in MATLAB (The MathWorks, Inc., Natick, MA, USA) at a sampling rate of 44.1 kHz with 16-bit resolution. The stimulus length was chosen to allow for head movements during playback, facilitating the use of dynamic binaural cues and thus enabling highest localization accuracy (Thurlow & Mergener, 1970).

### Virtual Sound Sources

Both spatial transfer function data sets, that is, HRTFs and HARTFs, used for the creation of VSSs were measured from an artificial head mannequin produced at the Institute of Technical Acoustics, RWTH Aachen University, with simple torso and detailed ear geometry (Minnaar, 2002; Schmitz, 1995). All filter sets had a length of 256 samples and were measured at a spatial resolution of 1° × 1° in azimuth and elevation. A detailed description of the spatial transfer function measurement procedure including an objective data analysis is provided in Pausch et al. (2018). The given spatial filter resolution lies well below or in the range of minimum audible angles reported by Mills (1958) and Perrott and Pacheco (1989). Depending on the experimental condition, the VSSs were generated by convolving the stimulus with the corresponding rendering filters, that is, HRTFs or HARTFs, using the real-time auralization software environment Virtual Acoustics (ITA Aachen, 2018; Wefers, 2015). For the selection of spatial rendering filters, a nearest-neighbor algorithm determined the filter subset based on the current real-world user position and orientation relative to the VSS. In case of loudspeaker-based playback, the user’s real-world position and orientation relative to the loudspeaker positions additionally trigger the selection of correct playback HRTFs. As filter exchange strategy, a time-domain cross fading technique was applied, enabling efficient time-varying filtering (Wefers, 2015). Assuming that listener movements exceed half of the spatial resolution of spatial transfer functions will result in maximum filter update rates of about 172 Hz, given an audio buffer size of 256 samples and a sampling frequency of 44.1 kHz.

### Experimental Design and Test Conditions

**Figure 1.**
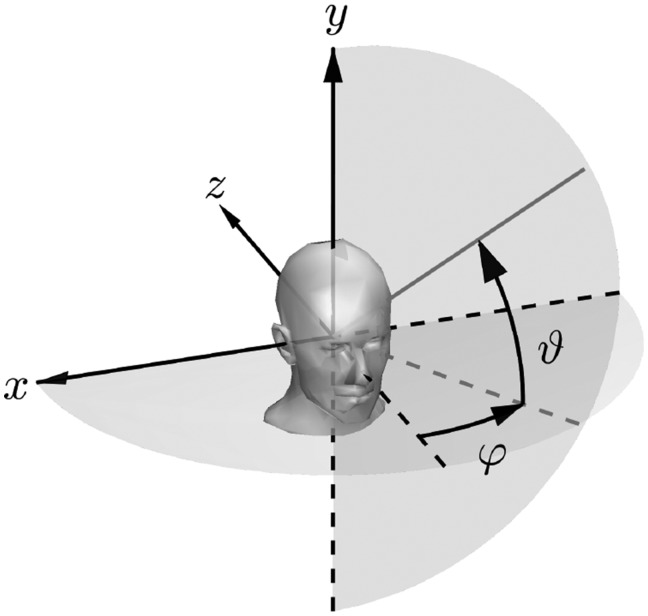
Definition of the Used Head-Related Spherical Coordinate System With Azimuth Angles φ and Elevation Angles ϑ.

In this article, a head-related spherical coordinate system is used, see Figure 1. By default, the listener looks in the negative *z*-direction. Azimuth angles increase counterclockwise and are represented by φ∈R|0≤φ<360, and elevation angles are represented by ϑ∈R|−90≤ϑ≤90, both provided in degrees.

In an open-loop sound localization task, the perceived directions of 12 sound sources, 8 of which were arranged in steps of $\varphi_{k}=k\cdot 45{}^{\circ}$, with k∈{0,1,…,7}, in the horizontal plane, and 4 sound source directions on the median plane at $\varphi_{0}$ and $\varphi_{4}$ and elevation angles of $\vartheta_{1,2}=\{30{}^{\circ},-30{}^{\circ}\}$, were tested. The presentation order was random while testing each source direction 3 times.

The study comprised two parts, each designed as a within-participant experiment and conducted on separate days, with two and three test blocks, respectively. To avoid first-order carryover effects, the block order was counterbalanced by means of a Latin square design. Both parts had one within-participant factor *System* with factor levels as described below. The first part was conducted in an anechoic chamber with the dimensions 9.2 m×6.2 m×5 m (L × W × H) to investigate localization of RSSs modeled by loudspeakers (level LS) and VSSs using headphones (level HP). For the second part, we used an acoustically optimized hearing booth (A:BOX, hearing test booth; Desone Modulare Akustik, Ingenieurgesellschaft mbH, Berlin, Germany) with the dimensions 2.3 m×2.3 m×1.98 m (L × W × H), fulfilling ISO 8253-1 (2010), ISO 8253-2 (2009), and ISO 8253-3 (2012); also cf. Pausch et al. (2018). Localization of VSSs reproduced over loudspeakers using CTC filters (level CTC), over research HAs (level RHA), and as combined over loudspeakers and CTC filters together with research HAs (level CTCwRHA) was assessed. These experimental conditions are summarized in Table 1.

**Table 1.** Summary of Experimental Conditions, Including the Listening Environments, Levels of the Within-Participant Factor *System*, Sound Source Types, and Playback Devices.

Index	Listening environment	Condition	Source type	Playback device
1	Anechoic chamber	LS	Real	Discrete loudspeakers
2	Anechoic chamber	HP	Virtual	Headphones
3	Hearing booth	CTC	Virtual	Loudspeakers with CTC filters
4	Hearing booth	RHA	Virtual	Research HAs
5	Hearing booth	CTCwRHA	Virtual	Loudspeakers with CTC filters and research HAs

*Note.* CTC = crosstalk cancellation; HA = hearing aid.

## 

### Apparatus

In condition LS, the stimuli were played back directly over one of the 12 two-way loudspeakers (Genelec 6010, Audio Export Georg Neumann & Co. GmbH, Heilbronn, Germany).

To minimize the influence of the headphone transducer characteristics on localization accuracy in condition HP, we applied robust headphone equalization (Masiero & Fels, 2011). Individual headphone transfer functions were measured 8 times, each time after repositioning the headphones (HD 600, Sennheiser, Wedemark, Germany), and applied as inverse filters, implemented as minimum-phase filters, on the respective binaural signal prior to playback.

For reproduction of VSSs over loudspeakers with CTC filters in condition CTC, four loudspeakers (K&H, O-110 Active Studio Monitor; Georg Neumann GmbH, Berlin, Germany) placed at $\varphi_{m}=m\cdot 45{}^{\circ}$, with m={1,3,5,7}, sharing a common elevation angle of ϑ=20° (Parodi & Rubak, 2010), were installed in the hearing booth. In combination with a four-CTC approach, driving all loudspeakers simultaneously, a robust binaural playback for all-around listener head rotations is possible (Lentz, 2008; Masiero, 2012). The CTC system matrix was optimal in the least squares sense with a Tikhonov regularization factor of 0.01.

In condition RHA, a custom-made pair of BTE receiver-in-the-ear research HAs without on-board digital signal processor (GN ReSound, Ballerup, Denmark), equipped with silicone ear pieces with holes, was used (cf., Pausch et al., 2018). Each research HA device had two omnidirectional micro-electro-mechanical system microphones (Knowles, Itasca, IL, USA). For this study, only HARTFs measured from the front microphones were used for the generation of VSSs since we did not simulate additional multichannel HA algorithms. No equalization was applied prior to playback as users would also listen to their acoustic environment in real life over HAs without additional equalization apart from the frequency-dependent gains or other spectral modifications caused by HA algorithms.

For a combined binaural reproduction in condition CTCwRHA, the signals of the research HAs were time delayed by 7 ms, relative to the loudspeaker-based reproduction (Stone et al., 2008). This relative delay was verified through artificial head measurements. The times of arrival were estimated by playing back an exponential sweep over a VSS and calculating the impulse responses accounting for the respective rendering and playback paths (Pausch et al., 2018).

**Figure 2.**
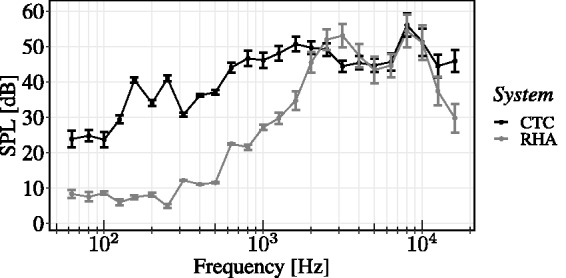
Contributions of Individual Reproduction Paths in Condition CTCwRHA to SPLs in Third-Octave Bands, Measured From the Right Ear of an Artificial Head With Ear Simulator and Averaged Across VSS Directions. Error bars represent 95% confidence intervals of the means. Note. SPL = sound pressure level; VSS = virtual sound source.

To prevent a bias due to level mismatches, playback levels in all experimental conditions of both experimental parts, see Table 1, were set to 65 dB(A) by means of calibrated artificial head measurements. In condition CTCwRHA, reproduction levels in both playback paths were matched (individual gains per path) while setting their combined playback level as done in the other experimental conditions (necessitating a combined gain of −3 dB). For further characterization of CTCwRHA, we measured in situ spectral sound pressure levels from an artificial head (HMS III, HEAD Acoustics, Herzogenrath, Germany) with ear simulator fulfilling ITU-T P.57 (2009) in two sequential measurement cycles. In the first one, the artificial head was placed in the center of the hearing booth at an ear height of 1.2 m to measure playback levels for all 12 VSS directions (condition CTC) but with attached research HAs and blocked ear canal (silicone ear piece with holes). In the second measurement cycle, we did the same for playback over research HAs alone (condition RHA). This helped us analyze the contributions of individual reproduction paths in condition CTCwRHA. Figure 2 shows measured sound pressure levels in third-octave bands with center frequencies between 62.5 Hz and 16000 Hz, averaged over all VSS directions. In condition RHA, distinct peaks at the first- and second-ear canal resonance frequencies and the typical spectral band limitation of the receiver response can be observed. Passive damping of the research HAs with open fitting becomes particularly relevant for frequencies and the peaks at the center frequencies of 155 Hz and 250 Hz between 2 and 8 kHz. Note that the spectral level decay toward lower frequencies in condition CTC can be attributed to the properties of HRTF magnitude spectra and the influences of the listening environment, respectively.

Two different six-degrees-of-freedom head-tracking systems were included to account for real-world user movements. Any translatory or rotational head movement influenced the selection of spatial rendering and playback transfer functions, as well as transfer paths for CTC filter calculations, and triggered virtual scene updates in real time. In the experiment’s first part, an electromagnetic tracking system (PATRIOT; Polhemus, Colchester, VT, USA) was used. According to manufacturer specifications, the system’s latency is 18.5 ms (Polhemus, 2018), while exhibiting static accuracy of 1.52 mm root mean square for three-dimensional (3D) position data, and 0.4° root mean square for sensor orientation data. No scientific investigation of this electromagnetic tracking system corroborating these data was found in the literature. Motion tracking in the second part relied on an optical tracking system (Flex 13, NaturalPoint, Inc. DBA OptiTrack, Corvallis, OR, USA). With an imager resolution of 1,280 × 1,024 (resulting in 1.3 MP) the system is able to resolve six-degrees-of-freedom tracking data in the submillimeter range. After system calibration, a summary assigned overall calibration results to the highest tier (“Exceptional”), acknowledging negligible mean 3D and two-dimensional reprojection and triangulation errors. Both tracking systems were set to the highest common tracker frame rate, which is, 60 Hz. For correct auralization, the offset of the rigid body, mounted on top of the participant’s head, to the center of the interaural axis was corrected individually. To the best of our knowledge, there is no scientific article on the latency of the applied optical tracking system using the exact same camera models and software version. However, Teather et al. (2009) reported latency values around 73  ±  4 ms for slightly different tracker hardware and settings (Flex:C120; 120  Hz frame rate; NaturalPoint, Inc. DBA OptiTrack). The corresponding author of Friston and Steed (2014) confirmed having used a different setup (Flex 3; 100  Hz frame rate; NaturalPoint, Inc. DBA OptiTrack) and reported mean latency values of 50.43 ms with a maximum value of 54.0 ms for the tested configuration “PC 3 OptiTrack Motive Rigid Body Aero Off” on a Windows 7 system. In combination with measured mean calculation times for the auralization of direct sound only (Pausch et al., 2018), dynamic end-to-end latency well below minimum detectable threshold values (Brungart et al., 2005; Lindau, 2009; Yairi et al., 2006) can be expected.

### Pointing Method

The indication of perceived sound source direction relied on an exocentric pointing method, as used by Richter and Fels (2016). On a display in front of the participant, a graphical user interface showed a sphere indicated by two great circles, the horizontal and the frontal plane, and an arrow in the center of the sphere depicting the participant’s virtual viewing direction. Using a game controller with two joysticks (Wireless Gamepad F710, Logitech, Romanel-sur-Morges, Switzerland), the participants were able to rotate a crosshair horizontally (gamepad’s right joystick) and vertically (gamepad’s left joystick) to mark the perceived sound source direction (gamepad’s green button). To support the 3D representation, the crosshair was additionally surrounded by a pursuant grid, spanning a spherical lune in the region of the crosshair’s direction, which was divided by squares of 5° × 5° each. The crosshair itself consisted of 20 vertical and 20 horizontal 1° × 1° squares, the center square marking the perceived sound source direction. To indicate sound source directions in the rear hemisphere, that is, the vertical hemisphere dividing the sphere by the frontal plane, the virtual viewing direction was invertible (gamepad’s blue button). Richter and Fels (2016) had reported a nonsignificant difference in pointing accuracy when using this method compared with nose pointing.

### Experimental Procedure

Both experimental parts started with the collection of informed consent and participant data. In the first part, individual headphone transfer functions were measured thereafter. Before each test condition, a training session, in which 10 sound source directions were tested, was conducted to familiarize the participants with the pointing method and the game controller. In these training sessions, the presented sound source direction was additionally marked as red square with the dimensions 10° × 10° on the sphere displayed in the graphical user interface. The participants had to point at one pixel within this red square and confirm its direction. For increased degree of difficulty, the red square’s surface gradually decreased to 1° × 1° in consecutive training trials. During the actual test session, no red square was shown. All source directions were tested randomly 3 times each. Participants were optionally allowed to repeat audio playback twice per trial (gamepad’s red button), effectively leading to 180 trials per participant for all conditions. To avoid fatigue, forced breaks of 5 min were included after each test block. In total, the experiment’s first and second parts took on average 45 min and 60 min, respectively.

### Reversal Rates

Since head movements likely shift the frontal plane relative to the presented static sound source directions, an adapted correction of reversal rates was applied. Similar to Chen (2003), individual localization trials were only corrected if the perceived azimuth angle lay within an angular range of ±30° around the presented azimuth angle mirrored on the frontal plane. In such a case, the perceived direction is mirrored on the frontal plane to the opposite hemisphere. Incorrectly located VSSs at $\varphi_{2}$ (90°) and $\varphi_{6}$ (270°) were not considered for correction due to the lack of definition. The reversal rate percentage per experimental condition was calculated by comparing corrected and uncorrected localization results, including the 10 relevant sound source directions.

### Angular Error Analysis

Three angular error metrics were introduced for assessing localization errors after correcting reversals: azimuth error $\epsilon_{\varphi }$, defined as the difference between presented and perceived azimuth angles, elevation error $\epsilon_{\vartheta }$, defined as difference between presented and perceived elevation angles, and overall error

(1) $$\epsilon_{\gamma }=\cos^{-1}[\text{cos(}\vartheta \text{)}\cos(\hat{\vartheta })\cos(\varphi -\hat{\varphi })+\sin(\vartheta )\sin(\hat{\vartheta })]$$

representing the great circle angle between presented and perceived sound source directions. $\varphi ,\;\hat{\varphi }$ and ϑ, $\hat{\vartheta }$ symbolize presented and perceived azimuth and elevation angles, respectively. Based on these definitions, the azimuth and elevation errors are always orthogonal to each other. All angular error metrics were evaluated in degrees and provided as absolute values, that is, as unsigned localization error, thus displaying error magnitudes.

### Hypotheses

The initial research questions led to two hypotheses, Hypothesis 1 (H1) and Hypothesis 2 (H2), as summarized in Table 2. All data analysis and statistical hypothesis testing presented below are based on a confidence level of 95%. When providing bootstrapped results, 10,000 bootstrap samples were used.

**Table 2.** Summary of Hypotheses.

Hypothesis	Prediction
H1	Compared with condition LS, performance decreases when localizing VSSs in conditions HP, CTC, RHA, and CTCwRHA.
H2	Compared with condition RHA, performance improves when localizing VSSs in condition CTCwRHA.

*Note.* VSS = virtual sound source.

## Results

### Reversal Rates

**Figure 3.**
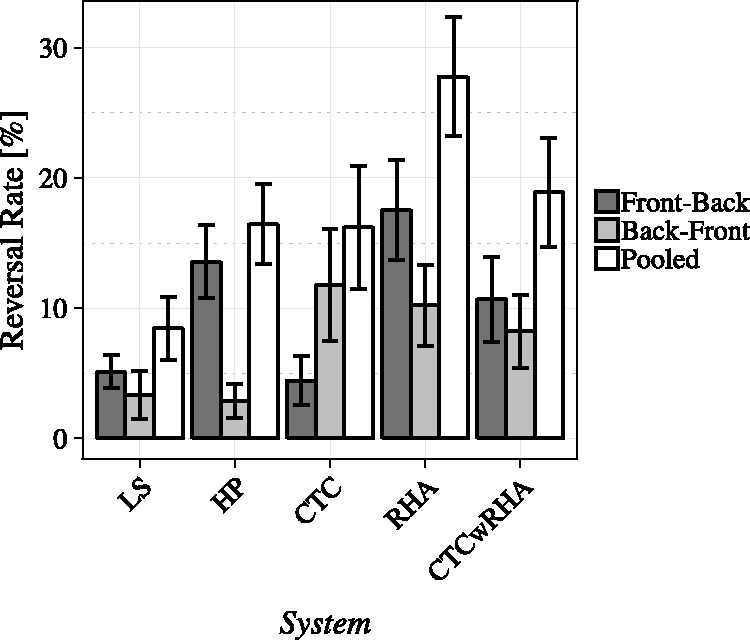
Mean Reversal Rates per Experimental Condition, Split Into Front-Back, Back-Front and Pooled Reversals. Error bars indicate one standard error of the mean.

Percentages of front-back, back-front, and pooled reversal rates are presented in Table 3 and Figure 3 per experimental condition.

**Table 3.** Summary of Performance Metrics.

Condition	Error metric					
	Reversal rate			Angular error metric		
	Front-back (%)	Back-front (%)	Pooled (%)	ϵ_φ_ (°)	ϵ_ϑ_ (°)	ϵ_γ_ (°)
	M ± SE	M ± SE	M ± SE	M ± SE	M ± SE	M ± SE
LS	5.1 ± 1.3	3.3 ± 1.8	8.4 ± 2.4	13.2 ± 6.2	11.3 ± 1.8	16.6 ± 2.3
HP	13.6 ± 2.8	2.9 ± 1.3	16.4 ± 3.1	17 ± 3.9	14.6 ± 1.4	23.6 ± 2.4
CTC	4.4 ± 1.9	11.8 ± 4.3	16.2 ± 4.7	21 ± 4.5	21.7 ± 1.6	29.1 ± 1.5
RHA	17.6 ± 3.8	10.2 ± 3.1	27.8 ± 4.6	29.6 ± 5.5	21.9 ± 2	39.3 ± 3.3
CTCwRHA	10.7 ± 3.7	8.2 ± 2.8	18.9 ± 4.2	20.3 ± 3.6	22.7 ± 2.2	33.8 ± 2

*Note.* Mean reversal rates with SEs, split into front-back, back-front, and pooled reversal rates, were calculated by comparing corrected and uncorrected perceived directions per system, including all participant trials. Angular error metrics, that is, azimuth error $\epsilon_{\varphi }$, elevation error $\epsilon_{\vartheta }$, and overall error $\epsilon_{\gamma }$, were evaluated based on the data averaged over participant trials. M = mean; SE = standard error.

Front-back confusions were the lowest for conditions LS and CTC with mean percentages of 5.1% and 4.4%, respectively, and increased substantially to 13.6% in condition HP. This trend continued to the highest average percentage of 17.6% in condition RHA and decreased to 10.7% in condition CTCwRHA.

Back-front confusion rates were lower than their front-back confusion counterparts in conditions LS, HP, RHA, and CTCwRHA with mean values of 3.3%, 2.9%, 10.2%, and 8.2%, respectively. Between conditions HP and CTC, this pattern is roughly inverted, the latter condition resulting in mean back-front percentages of 11.8%.

The lowest pooled average reversal rates of about 8.4% were observed in condition LS while increasing to 16.4% and 16.2% in conditions HP and CTC, respectively. On average, playback in condition RHA resulted in the highest pooled reversal rates of 27.8% and decreased to 18.9% in condition CTCwRHA.

### Overall Horizontal Source Localization

**Figure 4.**
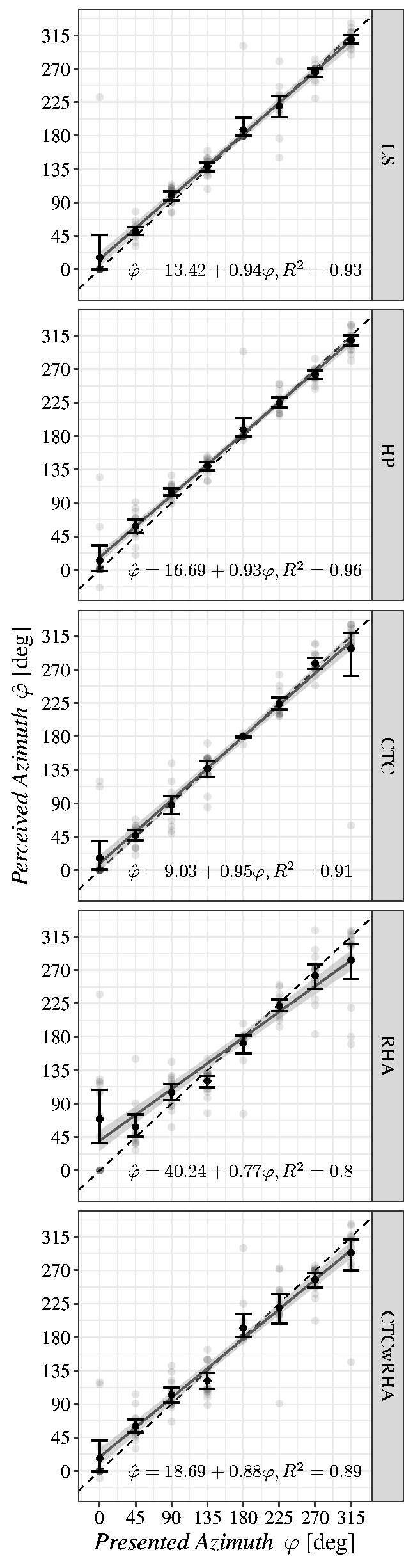
Corrected Localization Results per Experimental Condition for Source Directions in the Horizontal Plane Only. Regression lines are based on least squares regression including bootstrapped 95% confidence region. Black dots and error bars indicate means and their bootstrapped 95% confidence intervals, respectively, based on data points averaged across the three by-participant trials per source direction.

In order to analyze the overall horizontal source localization, source directions $\varphi_{k}$ in the horizontal plane were selected and consolidated by the factor *Presented Azimuth*. The results of each experimental condition (cf., Table 1) are shown as scatter plots with fitted linear regression lines in Figure 4. Each panel displays perceived and presented azimuth angles, ${\hat{\varphi }}_{k}$ and $\varphi_{k}$, on abscissa and ordinate in degrees, respectively, with corrected reversals. Dashed black lines represent perfect agreement of presented and perceived sound source directions. The perceived azimuth angles averaged over three trials per listener and source direction are indicated by gray data points. Gray linear regression lines represent the least squares fits of data points including the bootstrapped 95% confidence region. For all presented azimuth directions, means are drawn as black dots with error bars showing the bootstrapped 95% confidence interval of the mean. In addition, regression equations are provided including goodness-of-fit parameter *R*^2^. The data analysis aimed at detecting intercept and slope differences in regression line, facilitating the comparison of overall horizontal source localization performance across experimental conditions for hypothesis testing.

We formulated an LME model fit by restricted maximum likelihood estimation (Bates et al., 2015) with unconstrained and bounds-constrained quasi-Newton method optimizer (Nash, 2014; Nash & Varadhan, 2011) using R (R Core Team, 2019). The model predicted *Perceived Azimuth* based on crossed fixed-effect terms *Presented Azimuth*, recoded as numeric factor, and *System*, at the levels of experimental conditions, including the interaction term. The individual participant responses, averaged across trials, were entered as random by-participant intercepts. Two additional random-effect terms accounted for the nesting of participants within each level of *Presented Azimuth* and *System*. Two models of this type with identical structure were created to test the hypotheses. Model 1 referred to condition LS, allowing to compare intercept and slope to the ones observed in the remaining experimental conditions (H1). As we were also interested to see if the additional external sound field playback in CTCwRHA helped to improve the overall horizontal source localization, we referenced Model 2 to condition RHA, aiming at the detection of intercept and slope differences between conditions CTCwRHA and RHA (H2).

For the sake of a parsimonious model with minimized Akaike information criterion, we applied backward elimination on random-effect terms and subsequently on fixed-effect terms (Kuznetsova et al., 2017). Consecutive likelihood ratio tests on the random-effect structure suggested to drop nesting of participants within *System*, ${\text{χ}}^{2}(1)=0.067,p=.795$, the random by-participant intercept term, ${\text{χ}}^{2}(1)<0.001,\;p=.999$, while suggesting to preserve nesting of participants within *Presented Azimuth*, ${\text{χ}}^{2}(1)=37.27,\;p<.001$. *F* tests to investigate significant improvements in explained variance when dropping fixed-effect terms using Kenward–Roger’s method (Halekoh & Højsgaard, 2014) further proposed to include the interaction term *System* × *Presented Azimuth*, F(4,472)=8.89,p<.001. This backward elimination procedure improved the final model from an Akaike information criterion of 5,807.92 to 5,803.98. We checked the normality assumptions by visually inspecting standardized residuals versus fitted values, which did not reveal any obvious deviations. Accounting for the effect of clustering model terms, adjusted and conditional intraclass correlation coefficients (Nakagawa et al., 2017) of .2 and .02, respectively, motivated the use of an LME model with nested random effects. The final optimized model was able to explain approximately 90% of variance in *Perceived Azimuth*, reflected by marginal *R*^2^ considering fixed effects only, which significantly increased to 92%, reflected by conditional *R*^2^ considering both fixed and random effects.

The LME model coefficients of both variants of the final model are summarized in Table 4 (Lüdecke, 2018), showing good agreement with regression equations presented in Figure 4. For the calculation of *p* values, Kenward–Roger’s method (Kenward & Roger, 1997), implemented by Kuznetsova et al. (2017), was applied with subsequent Holm–Bonferroni correction (Holm, 1979).

**Table 4.** Summary of LME Model Coefficients, Fitting Horizontal Localization Results by Restricted Maximum Likelihood.

Coefficients	Perceived azimuth					
	Model 1 (re condition LS)			Model 2 (re condition RHA)		
	Estimate	95% CI [LL, UL]	*p*	Estimate	95% CI [LL, UL]	*p*
Fixed effects						
(Intercept)	13.42	[3.32, 23.51]	.066	40.24	[30.15, 50.34]	**<.001**
HP vs. LS	3.27	[−9.47, 16.01]	1.000			
CTC vs. LS	−4.39	[−17.13, 8.35]	1.000			
RHA vs. LS	26.82	[14.08, 39.56]	**<.001**			
CTCwRHA vs. LS	5.27	[−7.47, 18.01]	1.000			
* Presented Azimuth*	0.94	[0.88, 0.99]	**<.001**	0.77	[0.77, 0.82]	**<.001**
HP vs. LS × *Presented Azimuth*	−0.01	[−0.08, 0.06]	1.000			
CTC vs. LS × *Presented Azimuth*	0.01	[−0.06, 0.08]	1.000			
RHA vs. LS × *Presented Azimuth*	−0.17	[−0.23, −0.1]	**<.001**			
CTCwRHA vs. LS × *Presented Azimuth*	−0.05	[−0.12, 0.02]	0.774			
LS vs. RHA				−26.82	[−39.56, −14.08]	**.001**
HP vs. RHA				−23.55	[−36.29, −10.81]	**<.001**
CTC vs. RHA				−31.22	[−43.96, −18.48]	**.002**
CTCwRHA vs. RHA				−21.55	[−34.29, −8.81]	**<.001**
LS vs. RHA × *Presented Azimuth*				0.17	[0.1, 0.23]	**<.001**
HP vs. RHA × *Presented Azimuth*				0.16	[0.09, 0.22]	**<.001**
CTC vs. RHA × *Presented Azimuth*				0.18	[0.11, 0.25]	**<.001**
CTCwRHA vs. RHA × *Presented Azimuth*				0.11	[0.05, 0.18]	**.002**
Random effects						
σ^2^		760.56			760.56	
τ_00,*Presented Azimuth*:ID_		24.16			24.16	
Adjusted ICC/conditional ICC		0.2/0.02			0.2/0.02	
*N*_*Presented Azimuth*_		8			8	
*N*_ID_		15			15	
Model fit						
Number of observations		600			600	
Marginal *R*^2^/conditional *R*^2^		0.899/0.92			0.899/0.92	
AIC		5,803.98			5,803.98	

*Note.* Two models with identical structure and complexity were created, either referring to condition LS (Model 1) or condition RHA (Model 2). Mean coefficient estimates and their 95% CIs with lower and upper CI limits (LL and UL, respectively) are displayed with Holm–Bonferroni-corrected *p* values for fixed effects, which were calculated from *t* tests based on Kenward–Roger’s approximation for degrees of freedom. Bold *p* values represent statistically significant results at the 95% confidence level. Random factors are specified by the within-condition variance ${\text{σ}}^{2}$, the between-condition variance ${\text{τ}}_{00; Presented Azimuth\mathrm{:ID}}$ when nesting participants within *Presented Azimuth*, adjusted and conditional ICCs, the number of presented azimuth angles $N_{\mathrm{Presented}\;\mathrm{Azimuth}}$, and the number of participants $N_{\text{ID}}$. Information about the model fit is provided by the number of observations, marginal *R*^2^ (variance explained by fixed effects) and conditional *R*^2^ (variance explained by fixed and random effects), as well as the AIC value for both variants of the final model. CI = confidence interval; LL = lower level; LME = linear mixed-effects; UL = upper level; ID = participant identifier; ICC = intraclass correlation coefficient; AIC = Akaike information criterion; CTC = crosstalk cancellation; HA = hearing aid.

Post hoc tests in Model 1 revealed an intercept difference in RHA versus LS, t(472)=4.13, *p *<* *.001, and a slope effect of *Presented Azimuth*, t(505.83)=34.24, *p *<* *.001. These effects need to be interpreted in the presence of the significant interaction RHA versus LS × *Presented Azimuth*, t(472)=−4.82, *p *<* *.001, suggesting that the regression line slope in condition RHA, estimated at 0.77, is lower compared with condition HP, estimated at 0.93, thus partially supporting H1 (cf., Table 2) in terms of the overall horizontal source localization. No other significant effects or interactions were observed during post hoc analysis.

Post hoc tests in Model 2 resulted in an intercept effect of RHA, t(505.83)=7.81, *p *<* *.001, a slope effect of *Presented Azimuth*, t(505.83)=28.15, *p *<* *.001, and intercept differences in LS versus RHA, t(472)=−4.13, *p *<* *.001, in HP versus RHA, t(472)=−3.62, *p *<* *.001, in CTC versus RHA, t(472)=−4.8, *p *<* *.001, and in CTCwRHA versus RHA, t(472)=−3.32, *p *<* *.001. These effects need to be interpreted in the presence of significant interactions between LS versus RHA × *Presented Azimuth*, t(472)=4.82, *p *<* *.001, HP versus RHA × *Presented Azimuth*, t(472)=4.51, *p *<* *.001, CTC versus RHA × *Presented Azimuth*, t(472)=5.15, *p *<* *.001, and CTCwRHA versus RHA × *Presented Azimuth*, t(472)=3.34, *p *=* *.001. In terms of hypotheses testing, the interaction CTCwRHA versus RHA × *Presented Azimuth* suggested that the regression line slope in condition CTCwRHA, estimated at 0.88, was higher compared with condition RHA, estimated at 0.77, thus partially supporting H2 (cf., Table 2) regarding the overall horizontal source localization.

### Angular Error Analysis

**Figure 5.**
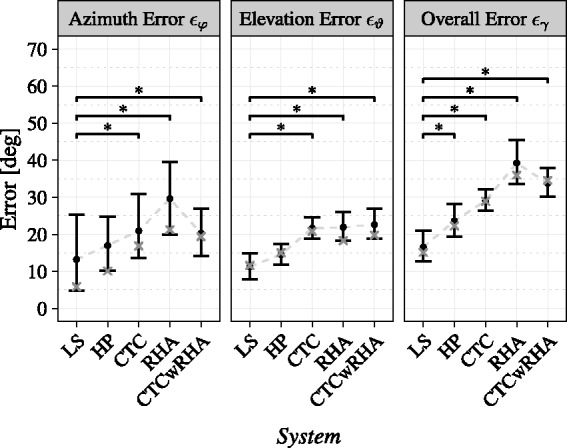
Unsigned Localization Errors per Experimental Conditions, Split Into Azimuth Error $\epsilon_{\varphi }$, Elevation Error $\epsilon_{\vartheta }$ and Overall Error $\epsilon_{\gamma }$. Black dots and error bars show means and their bootstrapped 95% confidence intervals, respectively, whereas crosses mark the median of averaged data over participant trials per experimental condition. Brackets with asterisks denote significant differences between experimental conditions at the 95% confidence level.

Angular error metrics, that is, unsigned azimuth error $\epsilon_{\varphi }$, elevation error $\epsilon_{\vartheta }$, and overall error $\epsilon_{\gamma }$, based on the localization results involving all 12 sound source directions, averaged per condition across participants, are presented in Figure 5 for all experimental conditions (cf., Table 1). Each panel displays one angular error measure for all experimental conditions, with black dots and error bars representing mean and bootstrapped 95% confidence intervals of the mean, respectively, and crosses marking medians.

Owing to the reduction of data complexity to clusters aggregating results of the corresponding angular error metrics per levels of *System*, the data analysis presented below is based on the analysis of variance (ANOVA). The Shapiro–Wilk test results suggested that for 90% of the log-transformed data, the residuals were normally distributed. As known to be robust against nonnormal data (Pearson, 1931; Schmider et al., 2010), we conducted three one-way repeated-measures ANOVAs, investigating the effect of the within-participant factor *System* on each angular error metric. For post hoc analysis, planned comparisons represented by the letter Δ with indices referring to experimental conditions (e.g., $\Delta_{21}$ representing the comparison between conditions HP and LS) were performed on log-transformed data using multiple *t*-tests with Holm–Bonferroni correction (Holm, 1979).

A one-way repeated-measures ANOVA with type III sum of squares revealed a significant effect of *System* on azimuth error $\epsilon_{\varphi },\;F(4,56)=7.88$, *p *<* *.001, $\eta_{\text{p}}^{2}=.36$. Planned comparisons with Holm–Bonferroni correction showed a significant increase of azimuth error in $\Delta_{31},\;t(56)=-3.83$, *p *=* *.002 (CTC, *M* = 20.95, standard error [*SE*] = 4.54; LS, *M* = 13.24, *SE* = 6.19), $\Delta_{41},\;t(56)=-5.29$, *p *<* *.001 (RHA, *M* =29.64, *SE* =5.51), and $\Delta_{51},\;t(56)=-3.72$, *p *=* *.002 (CTCwRHA, *M* =20.28, *SE* =3.58), thus partially supporting H1 in terms of azimuth error (cf., Table 2). No other significant differences were present, therefore not supporting H2 in terms of azimuth error.

A one-way repeated-measures ANOVA with type III sum of squares resulted in a significant effect of *System* on elevation error $\epsilon_{\vartheta },\;F(1.52,21.33)=8.44$, *p *=* *.004, $\eta_{\text{p}}^{2}=.38$. Mauchly’s test indicated that the assumption of sphericity had been violated, $\chi^{2}(9)=45.60$, *p *<* *.001; therefore, degrees of freedom were corrected using Greenhouse–Geisser estimates of sphericity, ε=.38. Planned comparisons with Holm–Bonferroni correction showed a significant increase of elevation error in $\Delta_{31},\;t(56)=-4.62$, *p *<* *.001 (CTC, *M* = 21.66, *SE* = 1.59; LS, *M* = 11.28, *SE* = 1.76), $\Delta_{41},\;t(56)=-4.56$, *p *<* *.001 (RHA, *M* = 21.93, *SE* =2.04.51), and $\Delta_{51},\;t(56)=-4.72$, *p *<* *.001 (CTCwRHA, *M* =22.66, *SE* =2.16), thus partially supporting H1 in terms of elevation error (cf., Table 2). No other significant differences were present, therefore not supporting H2 in terms of elevation error.

A one-way repeated-measures ANOVA with type III sum of squares revealed a significant effect of *System* on overall error $\epsilon_{\gamma },\;F(2.29,32.03)=21.23$, *p *<* *.001, $\eta_{\text{p}}^{2}=.60$. Mauchly’s test indicated that the assumption of sphericity had been violated, $\chi^{2}(9)=19.13$, *p *=* *.025; therefore, degrees of freedom were corrected using Greenhouse–Geisser estimates of sphericity, ε=.57. The results of planned comparisons with Holm–Bonferroni correction suggested a significant increase of overall error in $\Delta_{21},\;t(56)=-3.52$, *p *=* *.004 (HP, *M* =23.61, *SE* =2.39; LS, *M* =16.56, *SE* =2.32), $\Delta_{31},\;t(56)=-5.83$, *p *<* *.001 (CTC, *M* =29.09, *SE* =1.53), $\Delta_{41},\;t(56)=-8.2$, *p *<* *.001 (RHA, *M* =39.26, *SE* =3.25), and $\Delta_{51},\;t(56)=-7.08$, *p *<* *.001 (CTCwRHA, *M* =33.76, *SE* =1.95), thus partially supporting H1 (cf., Table 2) in terms of overall error. No other significant differences were present, therefore not supporting H2 in terms of overall error.

A summary of results from planned comparisons regarding angular error metrics between reproduction systems is provided in Table 5.

**Table 5.** Summary of Planned Comparisons Regarding Angular Error Metrics Between Reproduction Systems.

	Angular error metric		
Contrast	$\epsilon_{\varphi }$	$\epsilon_{\vartheta }$	$\epsilon_{\gamma }$
$\Delta_{21}$ (HP vs. LS)	*ns*	*ns*	*
$\Delta_{31}$ (CTC vs. LS)	*	*	*
$\Delta_{41}$ (RHA vs. LS)	*	*	*
$\Delta_{51}$ (CTCwRHA vs. LS)	*	*	*
$\Delta_{54}$ (CTCwRHA vs. RHA)	*ns*	*ns*	*ns*

*Note. ns =* nonsignificant at α=.05.

* Significant at α=.05.

## Discussion

### Reversal Rates

As expected, based on the results of previous studies, the lowest pooled average reversal rates were observed in condition LS (M=8.4%), potentially owing to the combined usage of individual static and dynamic binaural cues (Begault et al., 2001; McAnally & Martin, 2014; Thurlow & Runge, 1967). Similar reversal rates were reported in sound localization experiments with allowed head movements by Makous and Middlebrooks (1990), in which the presentation of broadband stimuli (bandpass filtered between 1.8 and 16 kHz) over loudspeakers with stimulus durations fixed at 150 ms (open-loop trials) was found to lead to a 6% reversal rate. This percentage is further supported by Wenzel et al. (1993) who presented trains of eight 250 ms bursts of Gaussian noise (bandpass filtered between 200 Hz and 14 kHz) with intermediate breaks of 300 ms, which resulted in reversal rates as low as 6.5%.

Although the reversal rates in condition HP (M=16.4%) were approximately twice as high as those in condition LS, they were substantially lower compared with results of headphone-based sound localization studies relying on static binaural synthesis with nonindividual HRTF data sets (e.g., Wenzel et al., 1993; M=31%). This performance difference is likely linked to a combination of mismatched spectral cues when using generic HRTFs and the absence of natural head movements. However, such head movements were identified to be among the most important cues for diminishing reversal rates in interactive binaural synthesis (Gilkey & Anderson, 2014; Oberem et al., 2018). Wenzel (1995) presented broadband Gaussian noise stimuli with a duration of 3 s via VSSs based on nonindividual HRTFs using dynamic binaural reproduction, and observed even lower front-back, although slightly higher back-front, confusion rates of 6.7% and 6.8%, respectively, compared with current results.

For condition CTC, average pooled confusions rates of 16.2% were also considerably lower than those reported by Takeuchi and Nelson (2002) who tested localization in a static CTC system, designed on the principle of optimal source distribution, which was set up in an anechoic chamber, also using generic HRTFs. Their testing procedure consisted of presenting VSSs with pink noise of a 3-s duration as source signal directly in front of the participant, followed by a VSS presenting 5-s pink noise, with a 3-s pause in between. Participants’ head movements were constrained using a headrest, resulting in average front-back and back-front confusion rates of 13.4% and 15.7%, respectively. Lentz (2008), however, stressed the importance of dynamic aspects and reported a substantial reduction of reversals in the interactive binaural auralization systems compared to the static variants. This notion was corroborated by perceptual experiments where VSSs were synthesized based on a two-loudspeaker CTC system playing back pulsed pink noise stimuli with 200 ms duration and successive 500 ms silence interval. Dynamic binaural synthesis outperformed its static counterparts in localization accuracy and reversal occurence, even in the presence of additional reflections emerging from three reflective walls added to the listening environment. Although this scenario is only roughly comparable to this study, with respect to the nature of the listening environment’s reflections and CTC implementation, the improvements in reversal rates observed in this study can be partially attributed to the supporting role of head movements. Interestingly, loudspeaker-based binaural reproduction with CTC filters seems to produce an inverse reversal pattern compared with headphone-based binaural reproduction, provoking more back-front than front-back confusions.

Apart from dynamic cues, another crucial factor related to the occurrence of reversals is linked to the monaural cues of HRTFs (Iida et al., 2007; Shaw, 2007) which, if distorted, can potentially increase the reversal rates (Oberem et al., 2018; Wenzel et al., 1993). An aggravated effect can be expected if these cues are reduced or completely absent when presenting VSSs based on HARTFs when measured using BTE HA devices (Denk et al., 2018; Kayser et al., 2009; Pausch et al., 2018; Thiemann & van de Par, 2019). As regards Figure 2, a substantially more influential factor could have been a lack of low-frequency energy in condition RHA. Owing to the transducer characteristics and the fitting type, the used research HAs show a strong negative sloping toward lower frequencies (Pausch et al., 2018), which particularly hinders conveyance of interaural time differences, in turn affecting horizontal source localization and thus reversal rates. Hebrank and Wright (1974) demonstrated that increasing the cutoff frequency when high-pass-filtering white noise leads to decreased localization ability on the median plane. To the best of our knowledge, there has been no localization experiment with bilateral HAs testing participants with NH on the basis of VSSs under free-field conditions for comparison purposes. Mueller et al. (2012) investigated localization performance in participants with NH, presenting everyday target stimuli via VSSs based on individually measured HARTF data captured by the microphones of BTE HA devices. These VSSs were presented in typical outdoor and indoor environments, simulated through image-source models and ray-tracing algorithms (Schimmel et al., 2009), applying binaural room impulse responses based on generic HRTFs. Participants were instructed to keep their head still while localizing the target stimuli reproduced via the receivers of completely-in-the-canal HA devices. When operated in omnidirectional mode, average front-back confusion rates of 43.1 ± 5.8% (*M* ± *SD*) across all VAEs were present, with three data sets lying in the range of chance level. As far as a comparison is possible, the decrease of reversal rates, as observed in condition RHA (pooled: *M* = 27.8%, front-back: *M* = 17.6%), might have been linked to the effect of head movements, as monaural cue distortion and missing interaural time differences were of comparable nature in both experiments. Deriving improvements solely from the effects of dynamic auralization might be misleading due to experimental setup differences and, of course, the influence of additional reflections. In order to disentangle these factors, specifically designed investigations need to be conducted. Besides, additional beamforming algorithms likely help to further decrease the reversal rates (Keidser et al., 2006; Mueller et al., 2012).

Presenting VSSs in condition CTCwRHA led to reductions in pooled reversal rates (*M* = 18.9%) compared with condition RHA. Participants with NH or mild HL are potentially susceptible to residual localization cues being transmitted through the open ear piece (Byrne et al., 1996). Additional binaural playback via loudspeakers and CTC filters enables listeners to make use of binaural HRTF cues, interaural time differences in particular, see Figure 2. Together with the precedence effect (Gardner, 1968; Litovsky et al., 1999), this combination seemed to have a positive effect on front-back and back-front confusion rates. Although additional playback via research HAs resulted in increased front-back and slightly decreased back-front reversal rates compared with what had been observed in condition CTC, the increase in pooled reversal rates was rather small.

### Overall Horizontal Source Localization

The potential perceptual differences leading to altered horizontal localization performance across experimental conditions using corrected localization results will be discussed in the context of spatial transfer functions, the used reproduction devices, and the listening environment.

Binaural listening was based on individual HRTFs (condition LS), generic HRTFs (conditions HP, CTC), generic HARTFs (condition RHA), or a mixture of HRTFs and HARTFs, both generic (condition CTCwRHA). Comparing the overall horizontal localization between LS and HP, we found the results from the LME model analysis to corroborate conclusions drawn by Wenzel et al. (1993) who stated that binaural cues are sufficiently maintained for a large part of listeners when reproducing VSSs based on generic HRTFs over headphones with a potential impact on front-back confusion rates. The performance in condition CTC seemed to be similar with respect to LS regarding nonsignificant differences in model intercepts and slopes. It was only the use of HARTFs in condition RHA, exhibiting substantial differences in binaural and monaural cues (Kayser et al., 2009; Pausch et al., 2018), that had an effect on overall horizontal source localization performance. In combination, these cue deviations seem to produce over- and underestimation of VSS directions in the first and fourth horizontal quadrants, respectively, while the effect of under- and overestimation in the second and third quadrants, respectively, is not so pronounced (cf., Figure 4 and Table 4). In the combined binaural playback in condition CTCwRHA, the overall horizontal localization of VSSs appeared to be dominated by cues similar to those available in condition CTC, rendering the overall horizontal localization performance comparable to that observed in condition LS. Analogous to inferences with respect to reversal rates, this improvement is potentially linked to mixed perception of additional low-frequency cues conveyed by the loudspeaker-based reproduction and the precedence effect.

As summarized in Table 1, various playback devices were used to model RSSs or reproduce VSSs. For condition RHA, spectral characteristics of the research HAs’ receivers were measured in Pausch et al. (2018), exhibiting distinct peaks at resonance frequencies of the used ear canal simulator (ITU-T P.57, 2009), see Figure 2. Considering only frequencies with spectral magnitude values of 30 dB below the peak value at around 2.6 kHz, the frequency range using an open fitting (silicone dome with holes) is bounded between 810 Hz and 15.4 kHz. In addition to inherent cue distortions of HARTFs, perceptual band limitation and the spectral receiver characteristics likely further mitigated the overall horizontal source localization performance. In CTCwRHA, however, reproduction over loudspeakers with CTC filters and low-frequency binaural cue restoration seemed to dominate perception, largely removing the detrimental effects on overall horizontal source localization linked to the receiver characteristics of the research HAs. Further investigations are necessary to determine how fitting gains and related HA algorithms will influence this positive effect of additional loudspeaker-based playback on overall horizontal source localization.

Localization in condition LS was measured under anechoic conditions, while performance in conditions CTC and CTCwRHA was assessed in a hearing booth (cf., Table 1). Given the negligible influence of the listening environment, other conditions are not addressed in this discussion. Localization experiments under free-field conditions with discrete loudspeaker playback typically assume negligible influence of additional reflections created by neighboring loudspeakers or the experimental hardware setup such as the loudspeaker mounting construction. Although it was originally claimed that binaural playback over loudspeakers and CTC filters also works best in anechoic conditions (Atal et al., 1966; Møller, 1992), Parodi and Rubak (2011) reported minimum channel separation for sufficient binaural signal perception. Based on the channel separation measured in the listening environment used for the second part of the experiment (Pausch et al., 2018), the implemented CTC system appeared to provide sufficient binaural cues for overall horizontal VSS localization as performance in CTC and CTCwRHA did not significantly differ from performance in LS. As a side note, we would like to add that the perceptual quality of acoustic CTC reproduction systems should not be judged only on the basis of channel separation but needs to take into account other factors such as spectral coloration (Choueiri, 2008), perceivable phase imperfections (“phasiness”), sweet spot sensitivity (Parodi & Rubak, 2010), and filter ringing or dynamic range overflow (Lentz, 2006).

### Angular Error Analysis

Evaluating the reproduction systems based on the introduced angular error metrics can be considered a refined analysis of performance differences between experimental conditions. Compared with overall horizontal source localization based on linear regression across horizontal VSS directions, angular errors per individual VSS directions in the horizontal and median planes were evaluated. Summaries of angular error metrics for all levels of *System* are provided in Table 3 and Figure 5 and are compared, as far as possible, to those from the literature.

For condition LS, the mean azimuth errors $\epsilon_{\varphi }$ lay within the open-loop unsigned horizontal error range of 1.5° to 15.9° for RSS localization at ϑ={65,85,95,115} reported by Middlebrooks and Green (1991). Elevation errors $\epsilon_{\vartheta }$ and overall errors $\epsilon_{\gamma }$ were in line and lower, respectively, when compared with results by Bronkhorst (1995). Results for $\epsilon_{\varphi }$ and $\epsilon_{\vartheta }$ in condition HP corroborated the findings of Begault et al. (2001) who had reported azimuth and elevation errors of 16.9 ± 7.8 (*M* ± *SD*) and 17.6 ± 14.6 (*M* ± *SD*) using headphone-based dynamic binaural synthesis based on generic HRTFs. Between the conditions HP and LS, error magnitudes differed significantly in terms of overall error $\epsilon_{\gamma }$ but not regarding azimuth error $\epsilon_{\varphi }$ and elevation error $\epsilon_{\vartheta }$. In contrast to overall horizontal source localization, this result partially confirms worse accuracy when localizing VSSs based on generic HRTFs compared with RSS, even when using headphone-based dynamic binaural reproduction (Bronkhorst, 1995).

Moving on to condition CTC, the overall error magnitudes $\epsilon_{\gamma }$ are surprisingly comparable to a reported average angle error (great circle angle) of 32.4° in localization experiments conducted by Gardner (1998), who had also used generic HRTFs but static binaural reproduction, while applying a band-limited, symmetric CTC system variant with an upper cutoff frequency of 6 kHz. The significant localization performance differences, as observed in all three angular error metrics compared with LS, suggest that the system imperfections discussed earlier were largely masked when analyzing overall horizontal source localization but became relevant when tailoring the analysis to the individual error angle components. These results indicate that condition CTC needs further optimization for accurate playback in nonideal listening environments (Kohnen et al., 2016; Sæbø, 2001), although the requirements concerning minimum channel separation were largely fulfilled (Parodi & Rubak, 2011; Pausch et al., 2018).

As far as we are aware, there are no matching results from the literature for comparison purposes of conditions RHA and CTCwRHA. Analogue to condition CTC, all three angular error components increased significantly. Potential reasons for this performance decrease have been discussed above with respect to reversal rates and overall horizontal source localization. What remains to be added is that both azimuth and elevation errors between conditions CTC and CTCwRHA were found to be very similar, which indicates that the monaural cue distortion in playback over research HAs is less consequential than the lack of low-frequency energy and decreased accessibility to interaural time differences.

Although we observed a positive effect of external loudspeaker playback between conditions CTCwRHA and RHA with respect to overall horizontal source localization, no such effect was seen in terms of angular error metrics. However, it should be noted that azimuth and overall error metrics suggested an insignificant trend towards localization improvement.

### Limitations of the Study

Although the output stream of the optical motion tracking system was used to update the virtual acoustic scene in real time, the motion tracking data were not recorded, thus preventing complementary analysis of natural head movements or possibly applied localization strategies. Therefore, it remains unclear whether the reduction of reversal rates in conditions HP, CTC, and CTCwRHA compared with static localization experiments in the literature can be attributed entirely to head movements. It is also conceivable that the generic HRTF data sufficiently matched the anthropometric data of certain participants, already lowering the reversal rates in case of relatively static listening. 

A condition where participants would listen to RSSs reproduced by loudspeakers by directly playing back time-delayed microphone signals over the research HAs’ receivers can be considered as the real-world equivalent to condition CTCwRHA. Such a condition would allow a comparison between real-world localization performance and the one in the VAE, facilitating further conclusions about effects related to generic and individual spatial transfer function data sets. However, in such a scenario, practical feedback issues need to be resolved by integrating a feedback cancellation algorithm whose behavior could affect RSS perception, possibly leading to biased results. A similar comparison therefore remains to be investigated as part of a specifically designed experiment on HA algorithms and their perceptual effects on selected spatial audio quality parameters. The finding that the localization performance of VSSs decreases in binaural loudspeaker playback, compared to RSS localization, raises the question whether the CTC setup in its current form, operated in nonideal listening environments, is adequately accurate. That said, it needs to be investigated whether the combined binaural reproduction approach is capable of sufficiently replicating the equivalent real-life listening situation using open-fit research HAs.

## Conclusions

We conducted a dynamic sound localization experiment to investigate differences in reproduction systems. The localization of RSSs modeled by discrete loudspeakers was compared with that of VSSs reproduced binaurally over headphones, loudspeakers with CTC filters, research HAs alone, or combined via loudspeakers with CTC filters and research HAs. We observed the highest reversal rates in playback over research HAs alone, most likely owing to missing binaural cues in lower frequencies, thus inhibiting sufficient access to interaural time differences given the spectral open-fit HA receiver characteristics. In combined reproduction, these missing cues could be partially restored, reducing the pooled reversal rates to those observed in binaural playback over headphones and loudspeakers. Compared with the results from static sound localization experiments using binaural VSS reproduction over headphones and loudspeakers, the dynamic binaural cues contributed to decreased reversal rates. The performance with respect to overall horizontal source localization in combined reproduction was similar to that when localizing RSSs while significantly improving compared with VSSs localization given playback over research HAs alone. Assessing the reproduction systems in terms of angular error metrics, including sound sources on the horizontal and median planes, the best localization accuracy could be attributed to VSS reproduction over headphones compared with RSS localization. Binaural reproduction over loudspeakers, combined via loudspeakers and research HAs and via research HAs alone, elicited inferior performance. In contrast to the improved overall horizontal source localization, additional binaural reproduction over loudspeakers did not significantly decrease angular errors. The results with respect to elevation errors and pooled reversal rates support the assumption that binaural cue restoration in combined reproduction was the main factor for improved localization, subordinating the influence of distorted monaural cues in HARTFs. Finally, the localization performance in combined reproduction can be considered as a baseline indicator for future experiments involving participants using open-fit research HAs, operated in omnidirectional mode.

## Data Availability

The authors had full access to all of the data in this study and take complete responsibility for the integrity of the data and the accuracy of the data analysis.
